# Heteroscedastic Bias-Robust Projected Gradient Descent for UWB Localization in Complex Indoor Environments

**DOI:** 10.3390/s26144578

**Published:** 2026-07-19

**Authors:** Zhongyang Yu, Qinghua Liu, Yong Qian

**Affiliations:** 1 School of Information and Communication, Guilin University of Electronic Technology, Guilin 541004, China; zyyu@mails.guet.edu.cn (Z.Y.); qhliu@guet.edu.cn (Q.L.); 2 Guangxi Baise Industrial Investment Development Group Co., Ltd., Baise 533000, China

**Keywords:** Ultra-wideband localization, indoor localization, NLOS mitigation, heteroscedastic noise, structural bias, projected gradient descent, robust optimization

## Abstract

Ultra-wideband (UWB) localization is widely used in indoor positioning because it provides high temporal resolution and direct geometric range constraints. In complex indoor environments, however, UWB ranging is affected by non-line-of-sight propagation, multipath reflection, heterogeneous measurement quality, and link-dependent persistent bias, producing long-tailed errors and trajectory drift. This paper proposes HBR-PGD, a heteroscedastic bias-robust projected gradient descent framework for UWB-only indoor localization. Its central idea is a role-separated error-source formulation that assigns packet-level quality degradation, anchor-channel persistent bias, and sparse instantaneous NLOS anomalies to different roles in a unified constrained residual model. Packet-level quality features are mapped to heteroscedastic uncertainty scales, robust loss shape parameters, and non-negative NLOS correction priors; anchor-channel soft gating limits residual-correction freedom; and target trajectories and bounded structural bias states are jointly estimated in overlapping sliding windows. Experiments on a public indoor UWB dataset show that HBR-PGD achieves RMSE values of 0.107 m, 0.068 m, and 0.204 m in residential-apartment, small-apartment, and workshop/industrial environments, respectively. Compared with WLS, MCC-VC-TOA, SR-MCC, and AR-PNN, HBR-PGD consistently improves overall accuracy and high-percentile robustness, with the largest gain in the workshop/industrial environment. Ablation results verify the contributions of heteroscedastic weighting, structural-bias estimation, gated correction, and information-weighted fusion. These results suggest that HBR-PGD is a practical UWB-only localization framework for complex indoor environments with heterogeneous measurement quality, persistent link bias, and sparse NLOS anomalies.

## 1. Introduction

Indoor localization is a fundamental enabling technology for intelligent logistics, warehouse management, industrial automation, mobile robot navigation, medical monitoring, emergency rescue, unmanned aerial vehicle control, and smart-building services [1,2,3]. In these applications, global navigation satellite systems are usually unreliable indoors because satellite signals are blocked, attenuated, or reflected by walls, floors, metallic structures, and large obstacles [1,4]. Achieving accurate and robust indoor localization under GNSS-denied or GNSS-degraded conditions therefore remains an important research problem for Internet-of-Things systems and intelligent cyber-physical systems.

Wi-Fi, Bluetooth Low Energy, radio-frequency identification, visible-light communication, inertial measurement units, visual-inertial odometry, and ultra-wideband (UWB) have all been widely investigated for indoor localization [1,2,5]. Among them, UWB has attracted considerable attention in high-precision indoor positioning and tracking systems because its large bandwidth, high temporal resolution, low power consumption, and strong multipath resistance can provide accurate time-of-flight or time-difference-of-arrival ranging information [2,6]. Compared with localization methods based on received signal strength, UWB ranging provides more direct geometric distance constraints and is therefore suitable for centimeter- or decimeter-level indoor positioning systems.

Nevertheless, UWB localization performance can degrade substantially in realistic, complex indoor environments. Under ideal line-of-sight (LOS) conditions, the signal propagation time approximately corresponds to the straight-line distance between the tag and the anchor. Under non-line-of-sight (NLOS) and multipath conditions, however, the direct path can be blocked by walls, furniture, human bodies, shelves, machinery, or metallic obstacles. The received signal may then arrive through reflected, diffracted, or obstacle-penetrating paths, increasing the propagation time and introducing ranging errors that are typically dominated by positive bias [7,8]. Such NLOS errors are often non-Gaussian, time-varying, spatially heterogeneous, and long-tailed, and they may markedly increase localization errors or even cause trajectory drift and localization failure in complex indoor scenarios [8,9].

Many approaches have been proposed for UWB localization under NLOS conditions, including NLOS identification, error compensation, and range-error mitigation strategies [8,10]. Classical geometric solvers, such as least squares, weighted least squares, Taylor-series methods, and Chan-type algorithms, are simple to implement and computationally efficient, but they are sensitive to biased and outlying range measurements [8,11,12]. Filtering methods, including Kalman filtering, extended Kalman filtering, unscented Kalman filtering, particle filtering, and adaptive robust filtering, can smooth localization trajectories through motion models and partially suppress random noise [11,13,14]. However, these methods usually depend on predefined system dynamics, noise covariance assumptions, or manually tuned threshold parameters. When the propagation environment changes rapidly, or when only some anchor links are affected by NLOS propagation, fixed noise assumptions cannot accurately describe the reliability differences among measurements.

Machine learning and deep learning methods have also been introduced into UWB indoor localization. One class of methods uses physical-layer features, such as channel impulse response (CIR), received power, first-path power, signal energy, and noise statistics, to identify LOS, NLOS, or multipath conditions [7,15,16]. Another class uses neural networks, recurrent models, attention mechanisms, or filtering-learning hybrid frameworks to compensate for ranging errors or directly estimate target positions from multi-source sensor data [5,6,17]. These methods have strong nonlinear modeling capabilities in complex indoor environments and are particularly suitable for propagation disturbances that are difficult to describe with conventional models. However, deep models usually require large training datasets; their cross-environment generalization can be affected by scene differences, and they may increase computational complexity. In addition, fusing UWB with IMU, vision, or other sensors can improve localization stability and NLOS resilience [5,6,13,18], but it also increases hardware cost, calibration difficulty, and deployment complexity.

In practical UWB systems with multiple anchors, multiple channels, and packet-level sampling, ranging error is rarely caused by a single noise source. Measurement quality can vary significantly across anchors, channels, and packets; some NLOS errors appear as slowly varying structural bias associated with specific anchor-channel links; and dynamic occlusions, abrupt changes in reflected paths, or hardware ranging mismatch can introduce sparse long-tailed outliers. Existing robust localization methods often suppress abnormal measurements through a single residual weight, a single robust loss, or a prior LOS/NLOS decision. Such treatments do not sufficiently distinguish error sources with different physical origins, time scales, and optimization roles. If packet-level uncertainty, persistent link bias, and instantaneous residual anomalies are all handled as the same type of disturbance, the localizer may still assign excessive confidence to low-quality measurements or incorrectly absorb persistent link bias into the estimated target position.

This paper proposes HBR-PGD, a heteroscedastic bias-robust projected gradient descent localization framework for complex indoor environments. The method uses only UWB range observations, synchronously acquired packet-level quality features, and known anchor coordinates; it does not rely on IMU, vision, or map information. The main contribution is a role-separated error-source formulation: packet-level quality controls residual reliability and tail behavior, anchor-channel bias states absorb slowly varying positive bias, and soft-gated non-negative correction priors suppress sparse instantaneous NLOS residual anomalies. These roles are then solved in a unified constrained sliding-window objective rather than being applied as independent sequential correction modules.

HBR-PGD is not intended as a simple combination of heteroscedastic weighting, robust loss, soft gating, and sliding-window optimization. Its novelty lies in assigning the dominant UWB ranging-error mechanisms to different mathematical roles in the same residual model. In packet-level UWB ranging, quality degradation mainly changes residual uncertainty, fixed anchor-channel propagation paths can generate slowly varying structural bias, and abrupt occlusions or reflection-path changes can produce sparse long-tailed anomalies. HBR-PGD models these effects through residual scale and loss-shape parameters, bounded structural-bias states, and gate-activated non-negative correction priors, respectively.

The main contributions of this paper are as follows:

1. A UWB-only role-separated error-source formulation is proposed. It decomposes complex indoor UWB ranging errors into packet-level heteroscedastic uncertainty, anchor-channel slowly varying structural bias, and sparse long-tailed NLOS residual anomalies and assigns them to distinct roles in a unified constrained residual model.
2. A quality-driven residual parameterization method is developed. It maps packet-level UWB quality features to heteroscedastic uncertainty scales, robust loss shape parameters, and non-negative correction priors, while group-level soft gating limits the degrees of freedom of instantaneous residual correction.
3. A bias-coupled constrained sliding-window PGD solver is designed to jointly estimate trajectory states and bounded structural-bias states in local windows. Overlapping-window estimates are then fused using information weights derived from local objective curvature.
4. The proposed method is evaluated against WLS, MCC-VC-TOA, SR-MCC, and AR-PNN under the same data protocol, together with NLOS-stratified analysis, component-level ablation experiments, and computational-cost evaluation.

The remainder of this paper is organized as follows. Section 2 introduces the system model, the overall HBR-PGD architecture, and the projected-gradient solver. Section 3 describes the public dataset, experimental scenarios, compared methods, and evaluation metrics. Section 4 reports localization results, NLOS robustness analysis, module diagnostics, and computational cost. Section 5 concludes the paper and discusses future work.

## 2. Materials and Methods

The proposed HBR-PGD framework consists of three coupled components. HBR-HQRM generates measurement-level robust parameters from packet-level UWB quality features. HBR-SRSC computes anchor-channel group-level soft-gate weights from initial geometric residuals and quality priors. HBR-BCTR jointly optimizes the target trajectory and slowly varying structural bias within sliding windows. Unlike methods that require an online learned classifier or the fusion of IMU/vision information, the main optimization variables of HBR-PGD include only the target position states and structural bias states. Quality-driven parameters and soft-gate weights are precomputed before the main optimization and remain fixed during PGD iterations.

This section first defines the packet-level UWB measurement model and unified residual form. It then introduces quality-driven robust parameter generation, group-level soft-gate computation, bias-coupled trajectory optimization, and the projected-gradient solution procedure. Finally, the window fusion strategy, complexity analysis, and implementation characteristics are presented.

### 2.1. System Model and Problem Definition

This paper considers two-dimensional planar UWB localization in complex indoor environments. A tag moves at discrete time steps t=1,2,…,T. The known coordinate of the *a*-th UWB anchor is denoted by $s_{a}\in R^{2}$, where a=1,2,…,A. In practical UWB systems, range observations at the same time step may come from multiple radio-frequency channels and multiple packet-level samples. The raw range data are therefore organized as a packet-level measurement tensor:

(1) $$Z=\{z_{tacp}|t=1,\ldots ,T;\;a=1,\ldots ,A;\;c=1,\ldots ,C;\;p=1,\ldots ,P_{tac}\}.$$

Here, $z_{tacp}$ denotes the range observation corresponding to the *p*-th valid packet on channel *c* from anchor *a* at time step *t*. The quality feature acquired synchronously with each range packet is denoted by $q_{tacp}\in R^{d_{q}}$; it may contain received-signal accumulation power, CIR energy, received-signal-strength differences, first-path shape statistics, and noise statistics. Prior studies have shown that CIR, first-path power, received power, and related physical-layer features can reflect LOS, NLOS, and multipath conditions, making them suitable inputs for measurement-quality modeling [7,8,16].

The true target position at time step *t* is denoted by $x_{t}\in R^{2}$, and the whole trajectory is $X={\left\{x_{t}\right\}}_{t=1}^{T}$. UWB ranging errors in complex indoor environments are usually produced by multiple mechanisms. We adopt the following measurement decomposition:

(2) $$z_{tacp}={∥x_{t}-s_{a}∥}_{2}+b_{tac}+\nu_{tacp}+o_{tacp},$$

where $∥x_{t}-s_{a}{∥}_{2}$ is the true geometric distance from the target to the anchor, $b_{tac}$ denotes an anchor-channel-related slowly varying link-structured bias, referred to as structural bias in this paper, $\nu_{tacp}$ denotes measurement-level heteroscedastic random perturbation, and $o_{tacp}$ denotes sparse outliers caused by instantaneous occlusion, abrupt reflection-path changes, or hardware ranging mismatch.

To handle structural bias and sparse outliers simultaneously, the main optimization residual is defined as

(3) $$e_{tacp}=z_{tacp}-{∥x_{t}-s_{a}∥}_{2}-b_{tac}-m_{tac}{\hat{\delta }}_{tacp}.$$

Here, ${\hat{\delta }}_{tacp}$ is the packet-level non-negative NLOS correction prior induced by the quality features, and $m_{tac}\in \left(0,1\right)$ is the anchor-channel group-level soft-gate weight. The product $m_{tac}{\hat{\delta }}_{tacp}$ is the gate-activated residual correction. It is a precomputed instantaneous NLOS correction prior given by packet-level quality features and is not optimized in the main stage. By contrast, $b_{tac}$ is a slowly varying structural bias estimated during sliding-window optimization. The former handles packet-level sparse anomalies, while the latter absorbs persistent link bias; they are distinguished by whether they participate in optimization and by the temporal smoothness constraint on the bias state.

It is important to emphasize that ${\hat{\delta }}_{tacp}$ and $m_{tac}$ are precomputed before the main optimization and remain fixed during PGD iterations. The main optimization variables are only the trajectory *X* and the structural bias $B=\{b_{tac}\}$.

Equation (3) separates the residual into three error-source roles. First, the packet-level scale and robust loss parameters control measurement reliability and tail response. Second, the optimized bias state $b_{tac}$ represents persistent anchor-channel bias with temporal structure. Third, the fixed product $m_{tac}{\hat{\delta }}_{tacp}$ acts as a gate-activated correction prior for sparse instantaneous NLOS anomalies. This separation reduces the risk that all range errors are absorbed into a single residual weight or directly into the trajectory state.

To avoid confusing the main optimization residual with the anomaly diagnostic residual, we further define an initial geometric residual $r_{tacp}$, which is used only for soft-gate precomputation in HBR-SRSC. The residual $e_{tacp}$ in Equation (3) is the main residual used in the unified objective:

(4) $$r_{tacp}=z_{tacp}-{∥{\bar{x}}_{t}-s_{a}∥}_{2},$$

where ${\bar{x}}_{t}$ is the geometric initial position obtained by a lightweight initial localizer. The residual $r_{tacp}$ is used only for anomaly diagnosis and soft-gate precomputation before the main optimization, whereas $e_{tacp}$ is used in the main objective and changes as the current position estimate $x_{t}$ and structural bias $b_{tac}$ are updated.

### 2.2. Overall Architecture of HBR-PGD

**Figure 1:**
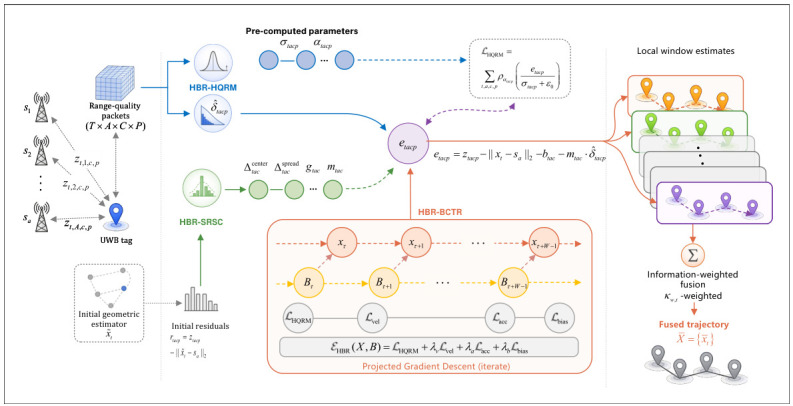
Overall architecture of the proposed HBR-PGD framework. Packet-level UWB range-quality observations and anchor coordinates are used as inputs. The quality-driven branch provides heteroscedastic uncertainty scales $\sigma_{tacp}$, robust loss shape parameters $\alpha_{tacp}$, and non-negative NLOS correction priors ${\hat{\delta }}_{tacp}$, while the residual-calibration branch computes group-level descriptors $\Delta_{tac}^{\mathrm{center}}$ and $\Delta_{tac}^{\mathrm{spread}}$, anomaly score $g_{tac}$, and soft-gate weight $m_{tac}$ from the initial geometric residuals. The trajectory and structural bias states are then jointly updated in overlapping sliding windows using projected gradient descent, and local window estimates are fused to obtain the final trajectory.

Figure 1 shows the overall data flow of HBR-PGD. The framework takes packet-level UWB range-quality observations and known anchor coordinates $s_{a}$ as inputs and produces a fused target trajectory as output. The purpose of this subsection is to summarize how the functional blocks are connected; the detailed parameter mappings, objective terms, and projected-gradient updates are given in the following subsections.

The component roles are summarized in Table 1.

The following subsections describe these components in order.

#### 2.2.1. HBR-HQRM: Heteroscedastic Quality-Robust Measurement

In HBR-PGD, HBR-HQRM constructs quality-driven measurement-level robust parameters. This component maps packet-level quality features to heteroscedastic scales, robust loss shape parameters, and non-negative correction priors, allowing different anchors, channels, and packets to have differentiated residual responses in the unified objective. First, the quality feature $q_{tacp}$ is standardized, and a composite quality score is computed as

(5) $${\tilde{q}}_{tacp}=w_{q}^{⊤}\mathrm{zscore}\left(q_{tacp}\right),$$

where $w_{q}\in R^{d_{q}}$ is the quality-feature combination vector determined by grid search on the calibration set. The heteroscedastic scale, robust loss shape parameter, and non-negative correction prior are then obtained through monotonic mappings:

(6) $$\sigma_{tacp}=\sigma_{\min}+\left(\sigma_{\max}-\sigma_{\min}\right)\mathrm{sigmoid}\left(-k_{\sigma }{\tilde{q}}_{tacp}\right),$$

(7) $$\alpha_{tacp}=\alpha_{\min}+\left(\alpha_{\max}-\alpha_{\min}\right)\mathrm{sigmoid}\left(k_{\alpha }{\tilde{q}}_{tacp}\right),$$

(8) $${\hat{\delta }}_{tacp}=\delta_{\max}\mathrm{sigmoid}\left(-k_{\delta }{\tilde{q}}_{tacp}\right).$$

Here, $\sigma_{tacp}$ controls the measurement-uncertainty bandwidth, $\alpha_{tacp}$ controls the tail behavior of the robust loss, and ${\hat{\delta }}_{tacp}$ is the non-negative NLOS correction prior. Because UWB ranging under NLOS conditions is usually characterized by path extension and positive range bias, setting ${\hat{\delta }}_{tacp}$ to be non-negative has a clear physical interpretation [8].

We use a generalized adaptive robust loss $\rho_{\alpha_{tacp}}\left(\cdot \right)$ to describe normalized residuals, following the adaptive robust loss family of Barron [19]:

(9) $$R_{\mathrm{HQRM}}=\sum_{t,a,c,p}\rho_{\alpha_{tacp}}\left(\frac{e_{tacp}}{\sigma_{tacp}+\epsilon_{0}}\right),$$

where $\epsilon_{0}>0$ is a numerical-stability constant and $\rho_{\alpha_{tacp}}\left(\cdot \right)$ is the generalized adaptive robust loss function. The scale $\sigma_{tacp}$ enters the denominator of the normalized residual directly, rather than acting as an additional multiplicative weight. This embeds measurement reliability into the loss geometry: when measurement quality is low, a larger $\sigma_{tacp}$ reduces the influence of that measurement on gradient updates; when measurement quality is high, a smaller $\sigma_{tacp}$ preserves its geometric constraint on the position. The parameter $\alpha_{tacp}$ further controls the tail response, allowing low-quality measurements to be more strongly suppressed in large-residual regions.

#### 2.2.2. HBR-SRSC: Sparse Residual Self-Calibration

To constrain the degrees of freedom of residual correction, HBR-PGD computes anchor-channel group-level soft-gate weights before the main optimization. HBR-SRSC constructs group-level anomaly scores from initial geometric residuals and quality-driven priors, and these scores regulate the influence of the non-negative correction prior in the unified residual. This module does not directly optimize $m_{tac}$; instead, it treats $m_{tac}$ as a fixed precomputed coefficient obtained from initial residual statistics, avoiding role confusion between gate variables and state variables.

First, the initial geometric residual $r_{tacp}$ is used to compute group-level robust statistics:

(10) $$\Delta_{tac}^{\mathrm{center}}={\mathrm{median}}_{p}\left(r_{tacp}\right),$$

(11) $$\Delta_{tac}^{\mathrm{spread}}={\mathrm{MAD}}_{p}\left(r_{tacp}\right).$$

Here, $\Delta_{tac}^{\mathrm{center}}$ describes the systematic offset of an anchor-channel group and mainly reflects systematic positive bias or persistent anomalies, whereas $\Delta_{tac}^{\mathrm{spread}}$ describes within-group measurement dispersion and mainly reflects range instability.

We further define the group-level residual correction prior as ${\bar{\delta }}_{tac}={\mathrm{median}}_{p}\left({\hat{\delta }}_{tacp}\right)$ and construct the composite anomaly score

(12) $$g_{tac}=\beta_{1}|\Delta_{tac}^{\mathrm{center}}|\;+\;\beta_{2}\Delta_{tac}^{\mathrm{spread}}+\beta_{3}\left|{\bar{\delta }}_{tac}\right|.$$

The non-negative weights $\beta_{1}$, $\beta_{2}$, and $\beta_{3}$ balance the effects of systematic offset, local dispersion, and the quality-driven prior. The soft-gate weight is defined as

(13) $$m_{tac}=\mathrm{sigmoid}\left(\gamma (g_{tac}-\tau_{g})\right)\in \left(0,1\right),$$

where γ controls the steepness of the gating curve and $\tau_{g}$ is the anomaly activation threshold. A value of $m_{tac}$ close to 1 indicates that the anchor-channel group is likely affected by severe NLOS propagation or long-tailed anomalies, so its residual correction prior is more fully activated in the main residual. A value close to 0 indicates that the group is relatively healthy, so the additional correction term has little influence on the main optimization.

#### 2.2.3. HBR-BCTR: Bias-Coupled Trajectory Refinement

During sliding-window optimization, HBR-PGD jointly includes the target trajectory state and the anchor-channel structural bias state in the optimization variables. HBR-BCTR describes this trajectory-bias coupling and constrains the local-window solution through motion smoothness and bias regularization. Unlike approaches that only smooth the trajectory, HBR-BCTR introduces the structural bias state $B=\{b_{tac}\}$ so that persistent NLOS positive bias can be absorbed by the bias state rather than being fully forced into the position estimate.

The trajectory smoothness term includes velocity and acceleration constraints:

(14) $$R_{\mathrm{vel}}=\sum_{t=2}^{T}{∥x_{t}-x_{t-1}∥}_{2}^{2},$$

(15) $$R_{\mathrm{acc}}=\sum_{t=3}^{T}{∥x_{t}-2x_{t-1}+x_{t-2}∥}_{2}^{2}.$$

The term $R_{\mathrm{vel}}$ suppresses excessive displacement between adjacent time steps, and $R_{\mathrm{acc}}$ suppresses second-order motion changes. These constraints do not force the target to follow a predefined trajectory; rather, they provide weak kinematic regularization under strong noise.

The structural bias $b_{tac}$ is slowly varying. Unlike sparse anomalies, structural bias is often caused by fixed walls, metallic equipment, or stable occlusions and should not be forced to zero by sparse regularization. We therefore use an amplitude regularizer and a temporal drift constraint:

(16) $$R_{\mathrm{bias}}=\sum_{t,a,c}b_{tac}^{2}+\eta \sum_{t=2}^{T}\sum_{a,c}{\left(b_{tac}-b_{t-1,ac}\right)}^{2}.$$

The first term prevents unbounded growth of the structural bias, and the second term constrains the same anchor-channel bias to vary slowly across adjacent time steps; η is the temporal smoothness strength. The state regularization term of HBR-BCTR is finally written as

(17) $$R_{\mathrm{BCTR}}=\lambda_{v}R_{\mathrm{vel}}+\lambda_{a}R_{\mathrm{acc}}+\lambda_{b}R_{\mathrm{bias}},$$

where $\lambda_{v}$, $\lambda_{a}$, and $\lambda_{b}$ control the strengths of velocity smoothness, acceleration smoothness, and bias regularization, respectively. These weights are determined during calibration or validation and remain fixed during testing.

### 2.3. Unified Objective and Projected Gradient Solver

Combining HBR-HQRM, HBR-SRSC, and HBR-BCTR, we construct the unified objective over the decision variables *X* and *B*:

(18) $$E_{\mathrm{HBR}}\left(X,B\right)=R_{\mathrm{HQRM}}+\lambda_{v}R_{\mathrm{vel}}+\lambda_{a}R_{\mathrm{acc}}+\lambda_{b}R_{\mathrm{bias}}.$$

Expanded explicitly, this objective is

(19) $$\begin{array}{cc} E_{\mathrm{HBR}}\left(X,B\right)= & \sum_{t,a,c,p}\rho_{\alpha_{tacp}}\left(\frac{z_{tacp}-{∥x_{t}-s_{a}∥}_{2}-b_{tac}-m_{tac}{\hat{\delta }}_{tacp}}{\sigma_{tacp}+\epsilon_{0}}\right)\\ \; & +\lambda_{v}\sum_{t=2}^{T}∥x_{t}-x_{t-1}{∥}_{2}^{2}+\lambda_{a}\sum_{t=3}^{T}{∥x_{t}-2x_{t-1}+x_{t-2}∥}_{2}^{2}\\ \; & +\lambda_{b}\left[\sum_{t,a,c}b_{tac}^{2}+\eta \sum_{t=2}^{T}\sum_{a,c}{\left(b_{tac}-b_{t-1,ac}\right)}^{2}\right]. \end{array}$$

In this objective, $\sigma_{tacp}$, $\alpha_{tacp}$, ${\hat{\delta }}_{tacp}$, and $m_{tac}$ are all precomputed constants; only *X* and *B* are optimized. Because UWB NLOS bias is typically positive, the structural bias is constrained to the physically feasible domain

(20) $$0\leq b_{tac}\leq b_{\max}.$$

To reduce the memory cost of global optimization over long sequences and improve local numerical stability, HBR-PGD solves the objective in overlapping local time windows. Let the window length be *W*, and let adjacent windows have a fixed overlap ratio. In each local window, the position trajectory and structural bias are updated alternately. Given the state $\left(X^{k},B^{k}\right)$ at iteration *k*, the position update is

(21) $$X^{k+1}=\Pi_{\Omega }\left(X^{k}-\mu_{x}\nabla_{X}E_{\mathrm{HBR}}\left(X^{k},B^{k}\right)\right),$$

where $\mu_{x}$ is the position-update step size and $\Pi_{\Omega }\left(\cdot \right)$ denotes projection onto the spatial feasible domain Ω. If no explicit map boundary is available, Ω can be obtained by expanding the anchor-deployment range. The structural bias state is then updated as

(22) $$B^{k+1}=\Pi_{\left[0,b_{\max}\right]}\left(B^{k}-\mu_{b}\nabla_{B}E_{\mathrm{HBR}}\left(X^{k+1},B^{k}\right)\right),$$

where $\mu_{b}$ is the bias-update step size and $\Pi_{\left[0,b_{\max}\right]}\left(\cdot \right)$ denotes elementwise clipping projection. This projection prevents structural bias from absorbing range residuals without bound and, thus, reduces the risk of unidentifiable drift between the geometric position and bias state. Local iterations stop when the maximum number of iterations is reached, the relative objective change falls below a threshold, or the gradient norm satisfies a stopping criterion.

Because adjacent sliding windows overlap, a time step *t* may receive several local position estimates from different windows. Direct averaging ignores local measurement quality and error curvature in different windows. HBR-PGD therefore uses information-weighted fusion:

(23) $${\hat{x}}_{t}=\frac{\sum_{w∋t}\kappa_{w,t}x_{t}^{\left(w\right)}}{\sum_{w∋t}\kappa_{w,t}},\;\kappa_{w,t}={\mathrm{tr}}_{+}\left(\Lambda_{t}^{\left(w\right)}\right)+\epsilon_{\kappa }.$$

Here, $x_{t}^{\left(w\right)}$ denotes the local estimate of time step *t* from window *w*, and $\kappa_{w,t}$ is the local information weight of that estimate. The weight is approximated from the local curvature of the objective with respect to $x_{t}$ at the convergence point. For numerical stability, the trace of the non-negative clipped curvature matrix is used, with a small constant $\epsilon_{\kappa }$. Consequently, high-quality local windows with mutually consistent multi-anchor observations and small residuals obtain larger fusion weights, whereas windows contaminated by severe NLOS propagation or long-tailed anomalies are automatically down-weighted.

### 2.4. Complexity and Method Characteristics

Within each local window, the main computational cost comes from packet-level residual evaluation, normalized robust loss computation, and gradient calculation. Let *W* denote the number of time steps in one sliding window, *A* the number of anchors, *C* the number of radio-frequency channels, *P* the average number of valid packets for each time-anchor-channel group, and *K* the number of PGD iterations used in one local window. With these definitions, the dominant single-window complexity of packet-level residual evaluation and gradient calculation is

(24) O(KWACP).

The computational scales of the bias regularizer and trajectory smoothness terms are approximately O(KWAC) and O(KW), respectively, and are usually lower than the packet-level measurement term. The overall complexity is therefore dominated by the measurement residual term. Because different sliding windows can be computed independently, the framework is suitable for offline batch processing and can also be extended to online localization with a sliding-buffer implementation.

HBR-PGD has three methodological characteristics. First, quality-driven parameters and soft-gate weights are determined before the main optimization, avoiding the introduction of additional gating variables or complex inner learning procedures in PGD. Second, structural bias is kept within a physically feasible range through projection constraints, reducing the risk of state drift in nonconvex optimization. Third, the sliding-window solution limits the size of each optimization problem, keeping the computational load controllable under multi-anchor, multi-channel, and packet-level observation conditions.

Compared with AR-PNN, HBR-PGD uses discrete projected gradient descent rather than continuous-time neural dynamics. The objective, projection constraints, and stopping rules therefore correspond directly to standard numerical optimization. HBR-PGD also uses packet-level UWB quality features to set the robust scale, loss shape, and NLOS correction prior. As a result, different anchors, channels, and packets can have different robust residual geometries at the same time step. In addition, the structural bias state $b_{tac}$ separates slowly varying NLOS bias from the geometric position state.

In summary, HBR-PGD is a unified optimization framework built around three sources of complex indoor UWB error: heterogeneous measurement noise, slowly varying structural bias, and sparse long-tailed anomalies. The following experiments use a public UWB indoor localization dataset to verify localization accuracy, long-tail error suppression, and computational efficiency under different NLOS intensities.

## 3. Experimental Setup

This section describes the experimental dataset, evaluation protocol, compared methods, implementation settings, and evaluation metrics. The experimental design focuses on three questions: whether HBR-PGD improves overall localization accuracy in indoor environments with different levels of complexity; whether HBR-PGD maintains robustness and compresses long-tailed errors under medium and high NLOS conditions; and whether the accuracy improvement is achieved with acceptable computational cost.

### 3.1. Dataset Description and Preprocessing

The experiments are conducted on the public Indoor UWB Positioning and Position Tracking Data Set released by Bregar [20]. This dataset is designed for evaluating range-based UWB indoor localization and trajectory-tracking algorithms. It contains UWB range observations, channel impulse response (CIR) information, anchor coordinates, and tag reference positions collected in real indoor environments. The original acquisition covers four indoor environments. The measurement system consists of eight fixed UWB anchors and one mobile tag. To eliminate the influence of varying human walking speed, equally spaced sampling points resembling human walking paths are predefined in each indoor environment, and multiple UWB range and CIR measurements are collected at each reference position.

The measurement system uses DecaWave/Qorvo DW1000 UWB transceivers integrated in DWM1000 modules. Ranging is performed using the asymmetric double-sided two-way ranging (ADS-TWR) protocol. Compared with simpler two-way ranging, ADS-TWR can partially reduce ranging errors caused by node clock and frequency drift. During data collection, for each tag reference position, the measurement program traverses eight anchors and six available UWB channels, collecting multiple packet-level measurements for each tag-anchor-channel combination. The dataset is therefore suitable not only for conventional range-based localization evaluation but also for the packet-level heteroscedastic modeling, anchor-channel bias estimation, and sparse gating diagnosis proposed in this paper.

Three representative environments are selected from the original dataset and denoted as Environment A, Environment B, and Environment C. Environment A corresponds to environment0 in the original dataset and represents a residential-apartment scenario. The exterior walls are made of brick, and the interior walls are mainly composed of plaster and insulation materials. This scenario contains room boundaries, wall occlusions, and local corners, making it suitable for evaluating localization performance in ordinary residential environments. Environment B corresponds to environment1 and represents a smaller apartment with a more compact floor plan and denser local turns. Environment C corresponds to environment2 and represents a workshop/industrial environment with many machines and metallic devices, stronger radio-frequency reflection, and more pronounced multipath and NLOS ranging. The dataset paper also notes that most devices in environment2 are metallic, making this environment more likely to produce strong reflections and extensive NLOS conditions.

These three environments span residential apartments, small apartments, and industrial workshops, covering propagation conditions from ordinary indoor occlusion to complex metallic reflection. They are therefore suitable for evaluating HBR-PGD under different NLOS intensities, anchor visibility conditions, and ranging-quality levels.

The original dataset provides both range values and physical-layer information related to UWB reception. In addition to the range value, the available quality-related fields include CIR-related quantities, received-signal-strength information, and first-path information. These quality-related fields are used to construct the packet-level quality feature vector for HBR-HQRM. Ground-truth tag coordinates are used only for performance evaluation and parameter validation and are not input to the localization algorithms during testing.

Table 2 summarizes the three selected experimental environments.

Before algorithm evaluation, raw range packets are first arranged according to trajectory index, anchor index, channel index, and packet number. Invalid and unavailable observations are removed according to the dataset labels and the evaluation protocol used in this paper. For each valid range packet, the corresponding quality features are standardized before being input to HBR-HQRM to reduce the influence of different physical units on the composite quality score. All compared methods use the same anchor coordinates, range observations, and reference trajectories to ensure fair comparison.

### 3.2. Compared Methods and Implementation Settings

To evaluate the effectiveness of HBR-PGD, five localization methods are compared: weighted least squares (WLS), maximum-correntropy variable-center time-of-arrival localization (MCC-VC-TOA) [21], squared-range maximum correntropy localization (SR-MCC) [22], adaptive robust projected neural network (AR-PNN) [11], and the proposed HBR-PGD. All compared methods are evaluated under the same data protocol. They use the same public UWB dataset, the same environmental sequences, the same anchor coordinates, the same valid range observations, the same preprocessing rules, and the same evaluation metrics. The ground-truth reference trajectory is used only for performance evaluation and is not input to any localization algorithm.

WLS is used as a classical range-based UWB localization baseline and represents conventional geometric localization without robust anomaly modeling or structural bias separation. This method establishes geometric distance residuals from range observations between the tag and multiple anchors and estimates the tag position through weighted least squares. WLS is simple to implement and has a low computational cost, but its performance is vulnerable to NLOS positive bias, strong multipath propagation, and long-tailed outlying ranges. Because WLS does not explicitly model anchor-channel bias or use packet-level quality features for heteroscedastic modeling, it is an appropriate basic reference for conventional geometric localization.

The comparison includes both a correntropy-based TOA baseline and a squared-range maximum-correntropy baseline. MCC-VC-TOA follows the maximum correntropy criterion with a variable center for TOA localization, in which the kernel center and bandwidth are used to improve robustness against NLOS-induced range errors [21]. SR-MCC follows a squared-range maximum-correntropy formulation for robust TOA-based localization in NLOS environments [22]. These baselines suppress abnormal ranges through correntropy-type residual criteria under the same ranging-observation protocol, but they do not use the packet-level heteroscedastic parameters, anchor-channel structural-bias states, or information-weighted window fusion used by HBR-PGD.

AR-PNN is used as the most closely related robust optimization baseline. It addresses UWB localization in mixed LOS/NLOS environments by introducing an adaptive robust loss function and solving a nonconvex constrained optimization problem with a projected neural network. An important feature of AR-PNN is that it does not require prior knowledge of the NLOS bias magnitude and does not rely on explicit LOS/NLOS classification; its robustness mainly comes from the adaptive loss function and the adjustment of the ranging-error upper bound [11]. AR-PNN is therefore suitable for evaluating whether HBR-PGD can further improve performance on top of adaptive robust loss modeling.

For AR-PNN, the reimplementation follows the published objective, projection constraints, and iterative solution procedure. To ensure an equivalent comparison, AR-PNN and HBR-PGD are evaluated on the same public dataset, the same environmental sequences, the same train/test split or validation protocol, the same anchor configurations, the same valid range observations, the same preprocessing rules, and the same evaluation metrics. Method-specific hyperparameters are selected only from the ranges recommended in the original paper or from the same validation protocol used for the proposed method. No baseline is tuned with disadvantageous settings, and the ground-truth trajectory is not used during testing except for final metric calculation.

The compared methods represent different robust-localization philosophies. WLS represents conventional geometric localization without explicit anomaly modeling. MCC-VC-TOA and SR-MCC suppress abnormal ranges through correntropy-type robust residual criteria, but they do not explicitly assign persistent link bias and instantaneous residual anomalies to different state roles. AR-PNN uses an adaptive robust loss and a projected-neural-network solver to handle bounded NLOS errors, but it does not model packet-level heteroscedasticity or anchor-channel structural bias as separate variables. HBR-PGD differs from these baselines by using a role-separated residual model: packet-level quality controls residual reliability and tail behavior, bounded anchor-channel bias states absorb persistent positive bias, and soft-gated non-negative correction priors suppress sparse instantaneous NLOS anomalies.

In the implementation of HBR-PGD, a lightweight geometric localizer first provides the initial trajectory. This initial trajectory is used only to compute the initial geometric residuals required by HBR-SRSC. HBR-HQRM then generates packet-level heteroscedastic scales, robust loss shape parameters, and non-negative NLOS correction priors from the standardized quality features. HBR-SRSC computes soft-gate weights for each anchor-channel group from group-level residual statistics and quality-driven priors. Finally, HBR-BCTR jointly optimizes the target trajectory states and structural bias states in overlapping sliding windows.

During the main optimization, all precomputed quantities, including heteroscedastic scales, robust shape parameters, correction priors, and soft-gate weights, remain fixed. The PGD solver updates only the target trajectory *X* and structural bias *B*. To avoid over-tuning for a single environment or trajectory, the parameters are fixed before testing and remain unchanged under the same evaluation protocol. The main implementation parameters of HBR-PGD are listed in Table 3.

### 3.3. Evaluation Metrics

The pointwise Euclidean distance error is used as the basic evaluation quantity. Let the estimated position of the *t*-th trajectory point be ${\hat{x}}_{t}$ and the ground-truth reference position be $x_{t}^{\mathrm{gt}}$. The localization error is defined as

(25) $$d_{t}={∥{\hat{x}}_{t}-x_{t}^{\mathrm{gt}}∥}_{2}.$$

Based on this pointwise error, RMSE, MAE, median error, high-percentile error, and maximum error are used for comprehensive evaluation. RMSE is defined as

(26) $$\mathrm{RMSE}=\sqrt{\frac{1}{T}\sum_{t=1}^{T}d_{t}^{2}}.$$

RMSE is more sensitive to large errors and is suitable for measuring the overall error energy and the influence of long-tailed anomalies on localization. MAE is defined as

(27) $$\mathrm{MAE}=\frac{1}{T}\sum_{t=1}^{T}d_{t}.$$

MAE reflects the average absolute localization deviation and is less sensitive to extreme outliers than RMSE. The median error $E_{50}$ is defined as the median of all pointwise errors and characterizes the main body of the error distribution:

(28) $$E_{50}={\mathrm{median}}_{1\leq t\leq T}\left(d_{t}\right).$$

To further examine long-tailed errors, we use the 90th percentile error and the 95th percentile error, denoted by $E_{90}$ and $E_{95}$:

(29) $$E_{90}=P_{90}\left({\left\{d_{t}\right\}}_{t=1}^{T}\right),\;E_{95}=P_{95}\left({\left\{d_{t}\right\}}_{t=1}^{T}\right).$$

Here, $E_{90}$ and $E_{95}$ denote the thresholds below which 90% and 95% of the trajectory-point errors fall, respectively. They reflect stability in the high-error region. The maximum error is defined as

(30) $$E_{\max}=\max_{1\leq t\leq T}d_{t}.$$

The metric $E_{\max}$ describes the most severe local failure. Because a single maximum error can be affected by local geometric degradation or individual measurement anomalies, the analysis mainly uses RMSE, MAE, $E_{50}$, $E_{90}$, and $E_{95}$ for overall judgment, rather than using $E_{\max}$ alone as the primary performance indicator.

In addition to numerical metrics, trajectory plots, error cumulative distribution functions (CDFs), pointwise error curves, NLOS-stratified statistics, and module diagnostic plots are used for visual analysis. Trajectory plots show the spatial consistency between the estimated and reference paths. CDF curves evaluate whether the error distribution shifts toward lower errors. Pointwise error curves identify local abnormal peaks. NLOS-stratified statistics test robustness under different occlusion intensities. Heteroscedastic-scale distributions and soft-gate heatmaps explain the internal behavior of HBR-PGD modules.

## 4. Results and Discussion

This section evaluates HBR-PGD against WLS, MCC-VC-TOA, SR-MCC, and AR-PNN under the protocol described in Section 3. The evaluation covers three representative environments: a residential apartment, a small apartment, and a workshop/industrial site. The analysis focuses on overall accuracy, error distributions, ablation results, NLOS-stratified robustness, diagnostic behavior, and computational cost.

### 4.1. Overall Localization Accuracy

Table 4 summarizes the overall localization accuracy of all compared methods. Conventional WLS is strongly affected by NLOS positive bias and long-tailed ranging anomalies, especially in Environment C. HBR-PGD obtains the lowest RMSE in all three environments, with values of 0.107 m, 0.068 m, and 0.204 m in Environments A, B, and C, respectively. The advantage is most pronounced in the workshop/industrial scenario. In this environment, HBR-PGD reduces RMSE by 50.1% compared with MCC-VC-TOA, 53.7% compared with SR-MCC, and 34.8% compared with AR-PNN. This result indicates that the proposed role-separated residual model is particularly useful under strong multipath and NLOS conditions.

**Figure 2:**
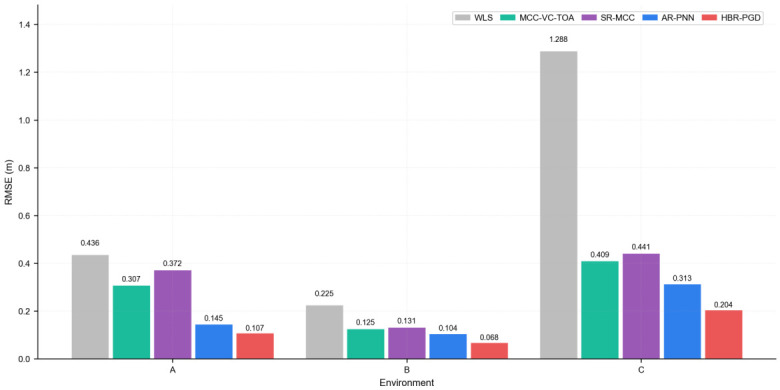
Overall RMSE comparison of WLS, MCC-VC-TOA, SR-MCC, AR-PNN, and HBR-PGD in the three evaluated environments. Lower values indicate higher localization accuracy.

Figure 2 provides a visual comparison of the RMSE values of all five methods across the three evaluated environments.

The high-percentile metrics show the same tendency. HBR-PGD obtains the lowest P95 error in all three environments, with values of 0.181 m, 0.118 m, and 0.341 m in Environments A, B, and C, respectively. These values are lower than those of all compared robust baselines. Therefore, the improvement is not limited to mean accuracy; it also compresses the high-error tail caused by NLOS and multipath effects.

HBR-PGD still shows a slightly larger maximum error than AR-PNN in Environment A. This indicates that individual trajectory points may remain vulnerable to instantaneous link anomalies or locally poor anchor geometry. For this reason, Max is treated as a supplementary indicator, whereas RMSE, MAE, median, P90, and P95 are used as the main performance criteria.

### 4.2. Trajectory-Level and Error-Distribution Results

**Figure 3:**
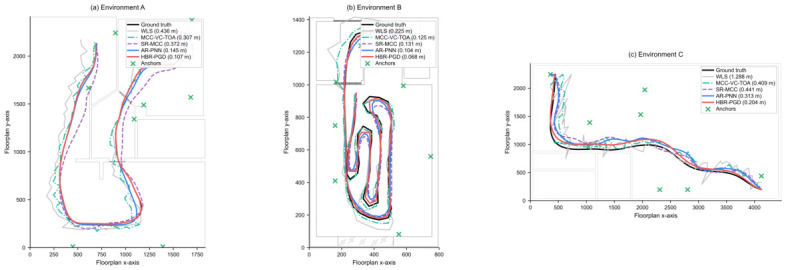
Trajectory comparison in the three evaluated environments. The figure shows the ground-truth trajectory and the estimated trajectories obtained by WLS, MCC-VC-TOA, SR-MCC, AR-PNN, and HBR-PGD. Environments A, B, and C correspond to the residential-apartment, small-apartment, and workshop/industrial scenarios, respectively.

Figure 3 presents trajectory-level comparisons in the three environments. Overall, WLS shows trajectory offsets of different degrees in all three environments, especially in Environment C. MCC-VC-TOA and SR-MCC reduce part of the WLS drift but still show local deviations in turning, occluded, or reflective regions. AR-PNN further improves trajectory stability in most segments. The estimated trajectory of HBR-PGD is generally closer to the ground truth, with a more continuous shape and fewer local jumps.

**Figure 4:**
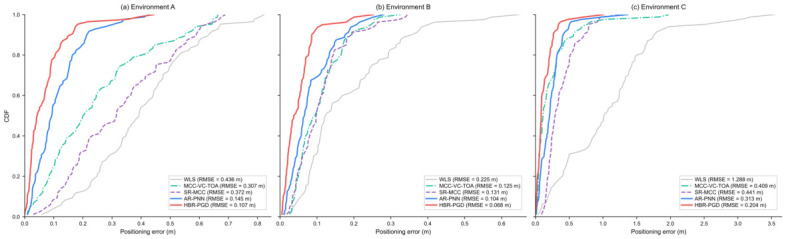
Cumulative distribution functions of pointwise localization errors in the three evaluated environments. The CDF curves compare the error distributions of WLS, MCC-VC-TOA, SR-MCC, AR-PNN, and HBR-PGD. A curve closer to the upper-left region indicates better localization performance.

Figure 4 presents the CDFs of pointwise localization errors. In all three environments, the HBR-PGD curve is closer to the upper-left region than the curves of WLS, MCC-VC-TOA, SR-MCC, and AR-PNN, indicating a higher proportion of small errors. The CDFs show that HBR-PGD shifts the error distribution toward smaller values and suppresses the high-error tail, especially in the workshop/industrial environment, where multipath and NLOS effects are strongest.

**Figure 5:**
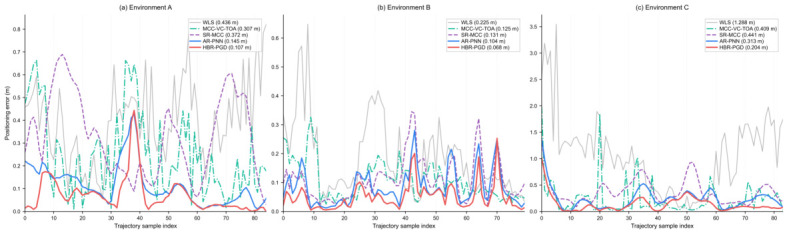
Pointwise localization error curves in the three evaluated environments. The figure plots the Euclidean positioning error along the trajectory index for WLS, MCC-VC-TOA, SR-MCC, AR-PNN, and HBR-PGD. Local peaks correspond to trajectory segments affected by strong NLOS, multipath, or geometric ambiguity.

Figure 5 further shows the pointwise localization error along each trajectory. WLS produces large error peaks in several segments, particularly in Environment C. MCC-VC-TOA and SR-MCC reduce some large deviations but still show intermittent error spikes. AR-PNN provides a more stable error curve than these robust residual baselines. HBR-PGD maintains the lowest error level over most trajectory points and suppresses most local error peaks, especially in the residential-apartment and workshop/industrial scenarios.

The pointwise error curves indicate that HBR-PGD cannot remove all local error peaks. Local failures may still occur when several anchor-channel groups are contaminated in the same window. They may also occur when the anchor geometry is insufficient for stable multilateration. This explains why HBR-PGD has a slightly larger maximum error than AR-PNN in Environment A, although it remains better in RMSE, MAE, median, P90, and P95.

### 4.3. Ablation Study

To verify that the proposed framework is not merely a combination of independent robustification techniques, we perform a component-level ablation study. The full HBR-PGD model is compared with four variants: w/o heteroscedastic weighting, w/o structural bias, w/o gated correction, and w/o information-weighted fusion. These variants correspond to removing the measurement-level reliability role, the persistent link-bias role, the sparse instantaneous residual-correction role, and the overlapping-window confidence-fusion role, respectively. In the variant without heteroscedastic weighting, all packet-level uncertainty scales are replaced by a constant scale. In the variant without structural bias, the anchor-channel bias state is removed by setting $b_{tac}=0$. In the variant without gated correction, the gate-activated correction term is removed by setting $m_{tac}{\hat{\delta }}_{tacp}=0$, so that sparse instantaneous NLOS correction is not used. This name is used because the setting removes the complete gate-activated correction term rather than the soft-gate weight alone. In the variant without information-weighted fusion, each trajectory state is estimated from a single local window without information-weighted fusion of overlapping estimates. All other settings, including the input data, initialization, robust loss configuration, optimization parameters, and evaluation metrics, are kept identical to those of the full HBR-PGD model.

Table 5 reports both RMSE and MAE results of the full HBR-PGD model and its ablation variants in the three environments. The full model consistently achieves the lowest error levels in both metrics, indicating that the improvement is not caused by a single isolated component. Removing heteroscedastic weighting increases both RMSE and MAE, showing that packet-level quality-dependent uncertainty modeling helps prevent low-quality measurements from being assigned excessive confidence. Removing the structural-bias state causes the most pronounced degradation in Environment C, which indicates that slowly varying anchor-channel bias is a major error source in the workshop/industrial scenario. The variant without gated correction also performs worse than the full model, confirming that the gate-activated non-negative correction prior is useful for suppressing sparse instantaneous NLOS residual anomalies. Removing information-weighted fusion further increases the localization error, showing that confidence-based fusion of overlapping window estimates improves trajectory stability. Overall, the RMSE and MAE ablation results support the non-redundant contribution of heteroscedastic weighting, structural-bias estimation, gated correction, and information-weighted fusion.

### 4.4. NLOS-Stratified Robustness Analysis

To further test whether the performance improvement of HBR-PGD comes from NLOS robustness rather than merely from trajectory smoothing, the errors are stratified according to the NLOS ratio of each trajectory point. The NLOS ratio denotes the proportion of valid observations marked as NLOS at that trajectory point. According to the empirical distribution of the NLOS ratio, samples are divided into Low-NLOS, Medium-NLOS, and High-NLOS subsets, and RMSE is computed for each subset.

**Figure 6:**
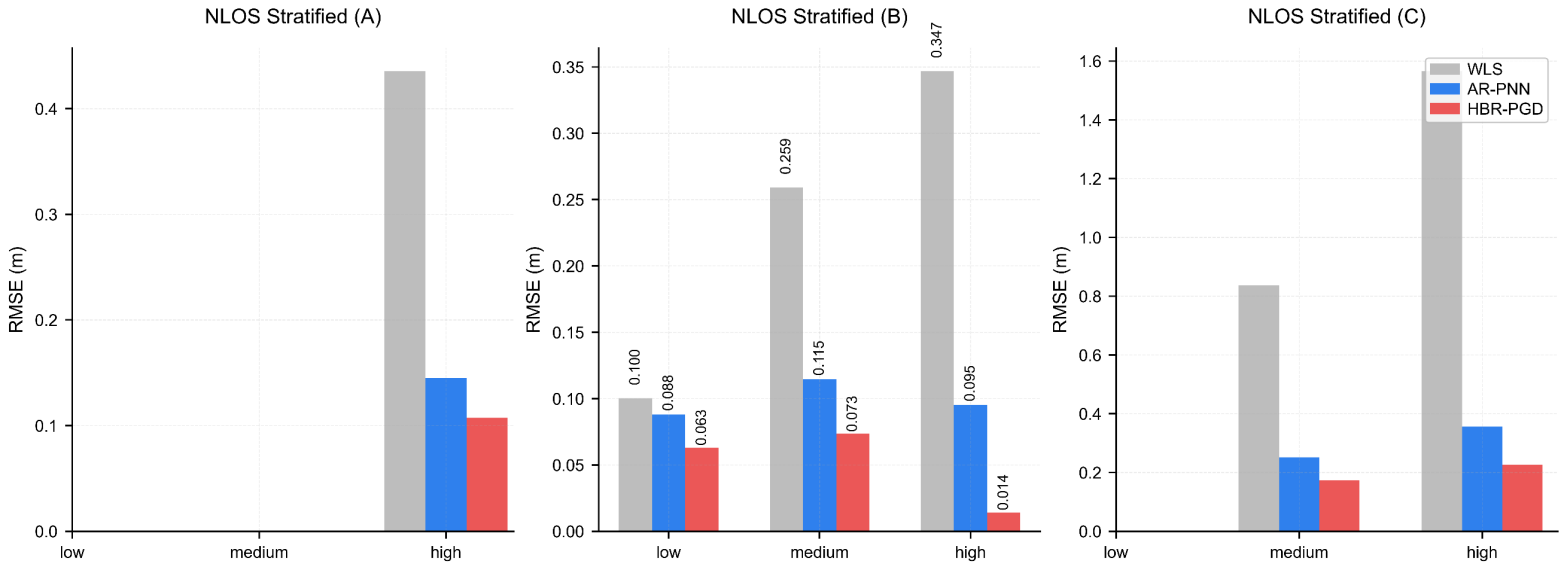
NLOS-stratified RMSE comparison. The samples are grouped according to their NLOS ratio, and RMSE is compared among WLS, AR-PNN, and HBR-PGD. The result indicates the robustness of HBR-PGD under different NLOS levels.

Figure 6 shows that the NLOS distributions differ significantly across environments. Environment A is a residential-apartment scenario, where internal walls and room boundaries cause many links to be occluded or partially occluded; thus, valid samples are more concentrated in the high-NLOS interval. Environment C is an industrial/workshop environment, where metallic equipment and complex reflective surfaces lead to fewer low-NLOS samples and make medium- and high-NLOS conditions more representative. This sample distribution is consistent with the physical characteristics of real UWB indoor deployments, where available ranging links in complex scenes are not uniformly LOS.

In Environment B, where the sample distribution is relatively balanced, HBR-PGD obtains the lowest RMSE in the Low-NLOS, Medium-NLOS, and High-NLOS subsets. In particular, under High-NLOS conditions, HBR-PGD maintains low error, whereas WLS and AR-PNN both degrade to different extents. This shows that HBR-PGD provides stable localization performance under different NLOS levels, especially under high NLOS conditions.

In the more representative industrial scenario, Environment C, the advantage of HBR-PGD is more pronounced. In the Medium-NLOS subset, HBR-PGD achieves an RMSE of 0.173 m, outperforming AR-PNN at 0.252 m and WLS at 0.837 m. Under High-NLOS conditions, where measurement anomalies fluctuate more strongly, HBR-PGD keeps RMSE at 0.227 m, whereas WLS rises to 1.566 m. This result indicates that conventional geometric localization is strongly affected by biased ranges under medium and high NLOS conditions, and it is consistent with the joint modeling of measurement quality, anomalous residuals, and structural bias in HBR-PGD.

These NLOS-stratified results show that the error reduction of HBR-PGD appears not only in global average metrics but also in robustness under medium and high NLOS conditions. Particularly in the industrial/workshop Environment C, WLS clearly degrades under high NLOS conditions, while HBR-PGD maintains low RMSE. It should be noted that NLOS-stratified results mainly reflect performance changes under different occlusion intensities; the internal mechanisms of each module are further analyzed through heteroscedastic-scale and soft-gate-weight distributions in the next subsection.

### 4.5. Parameter Behavior and Qualitative Interpretation

To further analyze the internal behavior of HBR-PGD, this subsection examines the parameter distributions of the complete framework under different measurement-quality and anchor-channel conditions.

**Figure 7:**
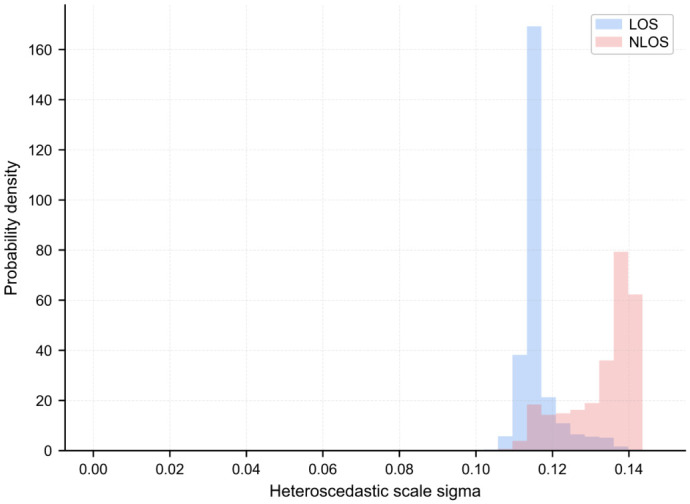
Distribution of heteroscedastic scale for LOS and NLOS measurements. NLOS measurements generally receive slightly larger uncertainty scales than LOS measurements, indicating different normalized residual responses under different measurement conditions.

Figure 7 shows the distribution of the heteroscedastic scale σ for LOS and NLOS measurements. The LOS/NLOS labels are used only for parameter-behavior interpretation and do not participate in the quality mapping of HBR-HQRM or the main optimization of HBR-PGD. NLOS measurements generally correspond to slightly higher σ values than LOS measurements. The statistics show that the mean σ is 0.117 for LOS measurements and 0.133 for NLOS measurements. This result is consistent with the expectation of quality-driven heteroscedastic modeling: when a measurement is more likely to be affected by occlusion, multipath, or reflection, a larger σ reduces its relative influence in the normalized residual; when measurement quality is high, a smaller σ helps preserve its geometric constraint.

The heteroscedastic scale σ is not a post-processing weight; it directly enters the residual-normalization denominator in the robust data term. Therefore, the distribution of σ assigned to different measurements affects the local residual response in the unified objective. The above results indicate that the heteroscedastic scale derived from quality features in HBR-PGD can distinguish LOS and NLOS measurements to some extent, providing parameter-level interpretation for measurement-quality modeling in the complete framework.

**Figure 8:**
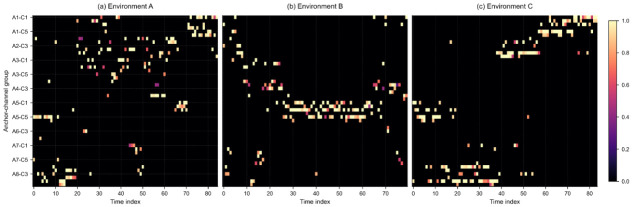
Soft-gate heatmaps of HBR-SRSC in the three evaluated environments. The gate values are not uniformly activated over all anchor-channel groups but tend to concentrate on specific time segments and link groups with higher residual inconsistency or lower quality priors.

Figure 8 shows the distribution of the soft-gate weight *m* in the three experimental environments. Overall, the soft-gate weights are not uniformly activated over all anchor-channel groups; instead, higher responses appear in specific time segments and anchor-channel combinations. This indicates that the group-level soft-gate parameters have nonuniform distributions in the experimental data. High values usually occur on links with larger initial residual offsets, higher within-packet dispersion, or poorer quality priors. This phenomenon is consistent with the design goal of HBR-PGD to restrict residual-correction freedom: the residual correction term is strengthened mainly in high-risk anchor-channel groups rather than applied equally to all measurements.

Across environments, soft-gate responses in Environment A mainly appear on links related to local occlusions and room boundaries, forming several discrete medium-intensity regions. Environment B has a smaller scene scale and a more compact trajectory; its soft-gate weights are generally sparse, indicating that most anchor-channel combinations still provide relatively stable geometric constraints. In contrast, Environment C shows stronger high soft-gate responses that are more easily concentrated on several persistent anchor-channel combinations. This agrees with the scenario characteristics of industrial/workshop environments, where metallic equipment is abundant, multipath propagation is stronger, and local NLOS conditions are more complex.

In summary, the distribution of the heteroscedastic scale σ and soft-gate weight *m* indicates that the precomputed parameters of HBR-PGD reflect measurement-quality differences and link-risk differences. These results provide qualitative support for understanding the behavior of the complete HBR-PGD framework in complex indoor environments.

### 4.6. Computational Cost Analysis

In addition to localization accuracy and robustness, computational cost is an important factor for evaluating the practicality of UWB indoor localization algorithms. The runtime comparison focuses on iterative robust localization methods with comparable trajectory-level optimization scopes, including SR-MCC, AR-PNN, and the proposed HBR-PGD. MCC-VC-TOA is not included in the main runtime figure because it is a lightweight epoch-wise TOA estimator and does not perform sliding-window trajectory optimization, structural-bias estimation, or information-weighted window fusion. Including it in the same bar plot would mix static per-epoch estimation with trajectory-level iterative optimization and would obscure the computational comparison among the main robust optimization baselines.

All runtime tests were conducted on the same computer equipped with an Intel Core i7-13700K CPU and 32 GB RAM, running Windows 11. All algorithms were implemented in Python 3.10. For each environment, every method was repeated 30 times under the same input data and parameter settings, and the reported runtime is the average over these runs.

Table 6 summarizes the runtime results of SR-MCC, AR-PNN, and HBR-PGD in the three evaluated environments.

**Figure 9:**
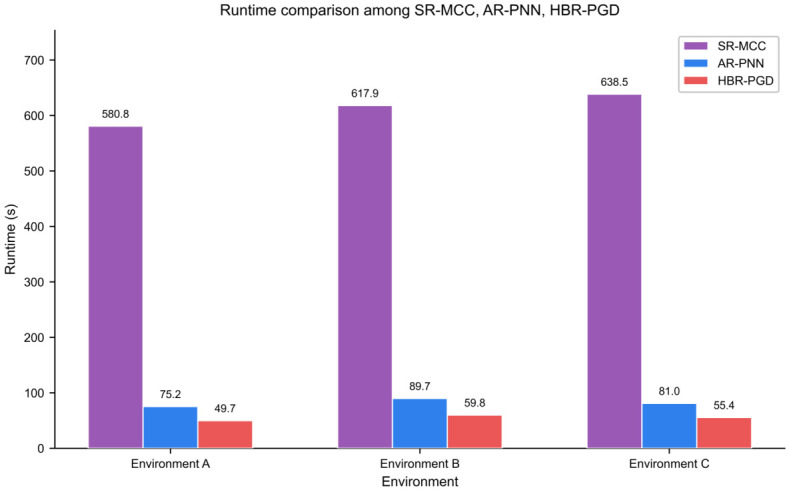
Runtime comparison among SR-MCC, AR-PNN, and HBR-PGD. The runtime results are averaged over 30 repeated runs under the same input data and parameter settings.

Among the trajectory-level robust optimization baselines included in the runtime analysis, HBR-PGD requires lower runtime than SR-MCC and AR-PNN in all three environments. Averaged over the three environments, HBR-PGD requires approximately 55.0 s, compared with 82.0 s for AR-PNN and 612.4 s for SR-MCC. This indicates that the proposed sliding-window PGD solver provides a favorable accuracy-efficiency trade-off among the robust optimization baselines evaluated in this paper. Figure 9 further shows the runtime comparison among SR-MCC, AR-PNN, and HBR-PGD. HBR-PGD is faster than both robust optimization baselines in all three environments, indicating that the proposed method maintains controllable computational overhead after introducing heteroscedastic robust modeling, soft-gated residual correction, and structural bias estimation. This result is related to the solution design of HBR-PGD: quality-driven parameters and soft-gate weights are precomputed before the main optimization and remain fixed during PGD iterations. The main optimization stage updates only the trajectory state and structural bias state, avoiding additional gate variables or complex inner learning procedures during iteration.

In addition, HBR-PGD uses overlapping sliding windows for local optimization rather than a one-shot global joint solution over the complete long sequence. This strategy limits the scale of each optimization problem, so the computational load in each local window is mainly determined by packet-level residuals and robust loss gradients. Combining this with the complexity analysis in Section 2.4, the dominant single-window complexity of HBR-PGD is approximately O(KWACP), where *K*, *W*, *A*, *C*, and *P* denote the number of PGD iterations, window length, number of anchors, number of channels, and average number of packets, respectively. Because different sliding windows can be processed independently, the framework also has potential for parallel implementation.

Together with the localization accuracy, error distribution, ablation, and NLOS-stratified results, the runtime results show that HBR-PGD improves localization robustness in complex indoor environments while maintaining an acceptable computational cost for offline or quasi-real-time UWB localization applications.

## 5. Conclusions

This study developed HBR-PGD for UWB-only indoor localization in complex indoor environments where ranging quality varies across packets, anchors, and channels. Across the three evaluated environments, HBR-PGD achieved the lowest RMSE and consistently reduced high-percentile errors compared with WLS, MCC-VC-TOA, SR-MCC, and AR-PNN. These results indicate that separating packet-level uncertainty, persistent anchor-channel bias, and instantaneous NLOS correction is effective for improving both average localization accuracy and the high-error tail.

From a practical perspective, HBR-PGD is most suitable for UWB deployments in which packet-level quality information is available but additional IMU, vision, or map constraints are difficult to introduce. The method uses existing UWB range-quality packets and known anchor coordinates. It can therefore improve trajectory stability without increasing hardware complexity. Its sliding-window PGD design and precomputed quality-driven parameters also make the method suitable for offline or quasi-real-time localization tasks in warehouses, industrial workshops, smart buildings, and other GNSS-denied indoor environments, especially when robustness is prioritized over a purely lightweight least-squares solution.

Several limitations remain. First, the evaluation is limited to two-dimensional single-tag localization on one public dataset and three selected environments. Second, the parameters are fixed after calibration, so performance may change when the hardware platform, anchor geometry, packet availability, or motion pattern differs substantially from the tested conditions. Third, HBR-PGD cannot fully eliminate local failures when several anchor-channel links are simultaneously affected by severe NLOS propagation or when the available anchors provide poor geometric constraints. Future work will validate the framework on larger-scale datasets, additional hardware platforms, dynamic multi-target scenarios, and three-dimensional localization tasks. It will also investigate online parameter adaptation, computational acceleration, and optional fusion with IMU, vision, or map constraints.

## Figures and Tables

**Table 1:** Component roles in the HBR-PGD framework.

Component	Input	Output	Role in Main Optimization
HBR-HQRM	Packet-level quality features	$\sigma_{tacp}$, $\alpha_{tacp}$, ${\hat{\delta }}_{tacp}$	Fixed robust residual parameters
HBR-SRSC	Initial geometric residuals and quality priors	$m_{tac}$	Fixed anchor-channel correction gate
HBR-BCTR	Range residuals and precomputed parameters	Local *X* and *B*	Optimized trajectory and structural-bias states
Window fusion	Overlapping local estimates and information weights	Final trajectory	Post-optimization aggregation

**Table 2:** Dataset statistics of the evaluated indoor environments.

Env.	Original	Scenario	Size	Points	Anch.	Ch.	Raw	Valid	Prot.
Environment A	environment0	Residential apt.	9.18×12.06 m	85	8	6	126,480	57,120	C
Environment B	environment1	Small apt.	3.60×6.69 m	80	8	6	119,040	53,760	C
Environment C	environment2	Workshop/industrial	21.96×11.85 m	84	8	6	124,992	49,392	M

Prot.: C = complete-anchor; M = missing-anchor-tolerant.

**Table 3:** Main implementation parameters of HBR-PGD.

Parameter	Meaning	Value
*W*	Sliding-window length	11
$\mu_{x}$	Step size for trajectory update	$1.0\times 10^{-3}$
$\mu_{b}$	Step size for bias update	$6.0\times 10^{-4}$
$\lambda_{v}$	Weight of velocity smoothness	0.02
$\lambda_{a}$	Weight of acceleration smoothness	0.04
$\lambda_{b}$	Weight of bias regularization	25.0
η	Temporal smoothness weight of bias	0.12
$\sigma_{\min},\sigma_{\max}$	Lower and upper bounds of heteroscedastic scale	0.052–0.200 m
$\alpha_{\min},\alpha_{\max}$	Lower and upper bounds of robust loss shape	[−1.5,1.8]

**Table 4:** Overall localization accuracy comparison among WLS, MCC-VC-TOA, SR-MCC, AR-PNN, and HBR-PGD.

Environment	Method	RMSE (m)	MAE (m)	Median (m)	P90 (m)	P95 (m)	Max (m)
Environment A	WLS	0.436	0.399	0.393	0.632	0.672	0.822
Environment A	MCC-VC-TOA	0.307	0.248	0.204	0.551	0.633	0.663
Environment A	SR-MCC	0.372	0.327	0.315	0.590	0.622	0.689
Environment A	AR-PNN	0.145	0.115	0.094	0.214	0.301	0.426
Environment A	HBR-PGD	0.107	0.072	0.044	0.158	0.181	0.443
Environment B	WLS	0.225	0.182	0.125	0.358	0.401	0.648
Environment B	MCC-VC-TOA	0.125	0.107	0.096	0.194	0.246	0.326
Environment B	SR-MCC	0.131	0.110	0.098	0.195	0.262	0.345
Environment B	AR-PNN	0.104	0.084	0.066	0.177	0.207	0.281
Environment B	HBR-PGD	0.068	0.051	0.040	0.086	0.118	0.253
Environment C	WLS	1.288	1.068	1.025	1.762	2.448	3.546
Environment C	MCC-VC-TOA	0.409	0.238	0.113	0.563	0.761	1.977
Environment C	SR-MCC	0.441	0.375	0.292	0.724	0.800	1.294
Environment C	AR-PNN	0.313	0.237	0.211	0.412	0.502	1.370
Environment C	HBR-PGD	0.204	0.136	0.084	0.265	0.341	1.010

**Table 5:** Ablation study results in terms of RMSE and MAE (m).

Environment	Metric	Full	w/o Het. Weighting	w/o Struct. Bias	w/o Gated Corr.	w/o Info. Fusion
Environment A	RMSE	0.107	0.126	0.133	0.130	0.135
Environment A	MAE	0.072	0.088	0.096	0.091	0.097
Environment B	RMSE	0.068	0.082	0.085	0.078	0.093
Environment B	MAE	0.051	0.064	0.067	0.060	0.073
Environment C	RMSE	0.204	0.238	0.288	0.265	0.272
Environment C	MAE	0.136	0.165	0.212	0.181	0.194

**Table 6:** Runtime comparison among SR-MCC, AR-PNN, and HBR-PGD.

Environment	Method	Total (s)	Per Step (ms)	Per Window (ms)
Environment A	SR-MCC	580.8	6833.4	34,167.2
Environment A	AR-PNN	75.2	884.8	4424.2
Environment A	HBR-PGD	49.7	584.7	2923.6
Environment B	SR-MCC	617.9	7724.3	38,621.4
Environment B	AR-PNN	89.7	1121.3	5606.3
Environment B	HBR-PGD	59.8	747.6	3738.0
Environment C	SR-MCC	638.5	7600.6	37,556.1
Environment C	AR-PNN	81.0	964.1	4763.6
Environment C	HBR-PGD	55.4	659.7	3259.6

## Data Availability

The public dataset analyzed in this study is the Indoor UWB Positioning and Position Tracking Data Set [20], published in *Scientific Data* and available at https://doi.org/10.1038/s41597-023-02639-5. Additional processed data and implementation details are available from the corresponding author upon reasonable request.

## Abbreviations

The following abbreviations are used in this manuscript:

ADS-TWR	Asymmetric double-sided two-way ranging
AR-PNN	Adaptive robust projected neural network
BCTR	Bias-coupled trajectory refinement
CDF	Cumulative distribution function
CIR	Channel impulse response
GNSS	Global navigation satellite system
HBR-PGD	Heteroscedastic bias-robust projected gradient descent
HQRM	Heteroscedastic quality-robust measurement
IMU	Inertial measurement unit
LOS	Line of sight
MCC-VC-TOA	Maximum-correntropy variable-center time-of-arrival localization
NLOS	Non-line-of-sight
PGD	Projected gradient descent
SR-MCC	Squared-range maximum correntropy localization
SRSC	Sparse residual self-calibration
TOA	Time of arrival
UWB	Ultra-wideband
WLS	Weighted least squares
